# Beyond graft function impairment after liver transplantation: the prolonged cold ischemia time impact on recurrence of hepatocellular carcinoma after liver transplantation—a single-center retrospective study

**DOI:** 10.7717/peerj.18126

**Published:** 2024-10-04

**Authors:** Jia Yu, Tang Yunhua, Yiwen Guo, Yuqi Dong, Jin long Gong, Tielong Wang, Zhitao Chen, Maogen Chen, Weiqiang Ju, Xiaoshun He

**Affiliations:** 1 First Affiliated Hospital, Sun Yat-sen University, Guangzhou, China; 2 Guangdong Provincial Key Laboratory of Organ Donation and Transplant Immunology, Guangzhou, China; 3 Guangdong Provincial International Cooperation Base of Science & Technology (Organ Transplantation), Guangzhou, China; 4 The First Affiliated Hospital of University of South China, Hengyang, China; 5 Hunan Provincial People’s Hospital, Changsha, China

**Keywords:** Gepatocellular carcinoma, Liver transplantation, Prolonged cold ischemia time, Tumor recurrence, Allograft dysfunction

## Abstract

**Purpose:**

Hepatocellular carcinoma (HCC) is one of the malignant tumors responsible for high mortality and recurrence rates. Although liver transplantation (LT) is an effective treatment option for HCC, ischemia-reperfusion injury (IRI) is a contributor to HCC recurrence after LT. Moreover, prolonged cold ischemia time (CIT) is a risk factor for IRI during LT, and there is insufficient clinical evidence regarding the impact of CIT on HCC recurrence after LT.

**Patients and Methods:**

This retrospective study analyzed 420 patients who underwent LT for HCC between February 2015 and November 2020 at The First Affiliated Hospital, Sun Yat-sen University. The duration of CIT was defined as the time from clamping of the donor aorta until portal reperfusion.

**Results:**

A total of 133 patients (31.7%) experienced tumor recurrence after LT, and CIT > 568 min was the independent risk factor for HCC recurrence (OR, 2.406; 95% CI [1.371–4.220]; *p* = 0.002). Multivariate Cox’s regression analysis revealed that the recipients’ gender, exceeding Milan criteria, poor differentiation, and alpha-fetoprotein (AFP) ≥400 ng/ml in CIT > 568 min group were independent risk factors for disease-free survival. The peak 7-day postoperative alanine aminotransferase (ALT) level (*p* < 0.001), the peak 7-day postoperative aspartate aminotransferase (AST) level (*p* < 0.001), the peak 7-day postoperative peak total bilirubin (TBIL) level (*p* = 0.012), and the incidence of early allograft dysfunction (EAD) (*p* = 0.006) were significantly higher in the CIT > 568 min group compared to the CIT ≤ 568 min group. Moreover, the amount of fresh frozen plasma (FFP) infusion during the operation increased (*p* = 0.02), and the time of mechanical ventilation postoperative was longer (*p* = 0.045).

**Conclusion:**

An effective strategy to improve the prognosis is to reduce CIT; this strategy lowers the recurrence of HCC in patients undergoing LT, especially those within the Milan criteria.

## Introduction

Hepatocellular carcinoma (HCC) is an aggressive tumor and the fourth leading cause of cancer-related death globally (Villanueva, 2019). More than 700,000 people die from HCC yearly. Theoretically, liver transplantation (LT) is an optimum treatment for HCC because it can eliminate both the tumor and underlying liver disease that progresses to cirrhosis. LT can eliminate the primary lesions, such as minute lesions that may be difficult to identify. It can also exterminate the possible cancerization of the rest of the liver. However, early HCC recurrence occurs after LT, which might result from micrometastasis when tumor cells are transported through the circulation (Choi et al., 2015). The 10-year recurrence-free survival rate after LT is around 50%–70% (Adam et al., 2018; Pinna et al., 2018). Prolonged waiting time is common among HCC patients diagnosed with LT, and this issue is a cause of tumor progression (Mehta et al., 2017).

The proportion of clinical use of extended criteria donors (ECDs) and the adoption of grafts with prolonged cold ischemia time (CIT) are increasing, considering the shortage of liver grafts, which may consequently lead to serious ischemia-reperfusion injury (IRI). Previous studies have demonstrated that prolonged cold ischemic time can greatly impair graft survival (Chini et al., 2021; Cassuto et al., 2008; Busuttil et al., 2005). Moreover, IRI has a positive correlation with HCC recurrence (Nagai et al., 2015; Grat et al., 2018). The use of ECD-derived grafts in patients with HCC can also accelerate tumor recurrence after LT (Chini et al., 2021; Cassuto et al., 2008; Busuttil et al., 2005). This retrospective study examined LT in patients with HCC to confirm the relationship between CIT and HCC recurrence.

## Patients and Methods

### Study population

This was a retrospective observational study of 508 patients with HCC (diagnosed by radiographic imaging and/or alpha-fetoprotein (AFP) levels, who presented for LT. From February 2015 to November 2020, the patients were admitted to the Department of Organ Transplantation; the First Affiliated Hospital, Sun Yat-sen University. Of 508 patients admitted, 88 patients were excluded because of the following issues: death within 30 days after the operation, nonprimary HCC confirmed by pathology, re-transplantation, pediatric liver transplant, and ischemic-free LT. All liver grafts were procured from donors after brain death or cardiac death. Informed consent to participate in the research was obtained. This study was approved by the Ethics Committee of the First Affiliated Hospital of Sun Yat-sen University (2022476), and was conducted in accordance with the principles of the Declaration of Helsinki. All the patients had signed human participant information consent. The duration of CIT was defined as the time from clamping of the donor aorta until portal reperfusion. Warm ischemia time (WIT) was calculated from the time when systolic blood pressure was <60 mm Hg and upon lying down (Kalisvaart et al., 2021). Immunosuppression was maintained with tacrolimus and mycophenolate mofetil. The recurrence of HCC was diagnosed by the abdomen and chest-computed tomography or magnetic resonance imaging and by AFP levels.

The recurrence time was calculated from the date of LT to the recurrence identification. Survival time was calculated from the date of LT to the death of the last follow-up period. Clinical characteristics of the donors included age, gender, donations after cardiac death (DCD), and laboratory data. Clinical characteristics of recipients included age, gender, comorbidity, indication for LT, laboratory data, model for end-stage liver disease (MELD) scores, treatment before LT contained neoadjuvant treatment, transarterial chemoembolization, radiofrequency ablation, and hepatectomy. Tumor information included pretransplant AFP level, tumor size, number of the tumors, microscopic vascular invasion, and tumor grade. Operative factors comprised operative time, volume of packed red blood cells (PRBC) transfusion, intraoperative blood loss, duration of anhepatic phase, CIT, and WIT. Post-operative factors included early allograft dysfunction (EAD), biliary complications, and acute rejection. The EAD is defined as the bilirubin level ≥10 mg/dL on day 7, international normalized ratio ≥1.6 on day 7, and alanine aminotransferase (ALT) or aspartate aminotransferase (AST) level >2000 IU/L within the first 7 days after LT, as proposed by Olthoff et al. (2010). According to clinical relevance, AFP threshold levelwere set to 400 ng/ml.

### Statistical analysis

Continuous variables that followed the normal distribution were expressed as means ± standard deviations and were compared using independent-samples *t*-tests. Moreover, median and interquartile ranges were compared using the Mann–Whitney *U* test for the 2-group. The Chi-square test was used to compare categorical variables given as numbers and percentages. Receiver operating characteristic (ROC) curves were plotted to determine the optimal cutoffs of continuous factors in predicting recurrence. A logistic regression model was used for multivariate analysis. The recurrence-free survival rate was calculated from the date of LT to the date of tumor recurrence. The data were right-censored if no evidence was found to confirm tumor recurrence on the day of the last follow-up. Multivariate analysis was used to identify significant variables in the univariate analysis of recurrence. All analyses were performed using SPSS software for Windows (version 26.0, SPSS Inc, IBM Corp, Chicago, IL, USA), and the level of significance was set at *p* < 0.05.

## Results

### Overview of demographic characteristics

A total of 420 LT cases that met the inclusion criteria were analyzed (Table 1). The donors were 315 male (75.0%), and their mean age was 36.98 ± 14.35 years. The majority of donors originate from donation after brain death donors (DBD), while donation after cardiac death (DCD) donors account for 7.1% of the total with a count of 30. Median levels of AST, ALT, and TBIL before harvest were 59 U/L (interquartile range: 30–106.99), 52.5 U/L (interquartile range: 27–95.5), 16.8 umol/L (interquartile range: 11.23–29.00), respectively. The median CIT was 418.5 min, and the median WIT was 5 min. The median age of the recipients was 52.3 (range of 21–75), and more than half of the recipients were male (52.1%, *n* = 219). The median of the last AFP level before LT was 29.17 ng/ml (interquartile range: 5.26–541.65). In addition, 281 recipients (66.9%) exceeded the Milan criteria. The mean size of the tumor was 53.21 mm (SD ± 40.23), and the majority of patients (84.3%) had been diagnosed with the hepatitis B virus. The mean of the MELD score was 14.57 (SD ± 40.23). Median AST, ALT, and TBIL peak levels in 7-day postoperative were 435 U/L (interquartile range: 232.75–993.25), 653 U/L (interquartile range: 345.25–1,023.5), and 105.45 umol/L (interquartile range: 60.235–174.075), respectively. The incidence of EAD was 151(36.0%).

**Table 1:** Overview of patients included in the study (*n* = 420).

	Number (%) or median (IQR)
Donor characteristics	
Age (years)	36.98 ± 14.35
Body mass index (kg/m^2^)	22.95 ± 18.30
Gender (male)	315 (75.0%)
Warm ischemia time(minutes)(80)	5 (5–8)
Cold ischemia time (min)	418.5 (351.5–522.25)
Sodium level before harvest(mmol/L)	153.77 ± 69.98
ALT level before harvest(U/L)	52.5 (27–95.5)
AST level before harvest(U/L)	59 (30–106.99)
TBIL level before harvest(umol/L)	16.8 (11.23–29.00)
DCD	30 (7.1%)
Recipient characteristics	
Age (years)	52.28 ± 10.21
Body mass index (kg/m^2^)	23.27 ± 3.31
Gender (male)	367 (87.4%)
Complications	
Hypertension	56 (13.3%)
Diabetes	54 (12.9%)
Coronary heart disease	9 (2.1%)
MELD	14.57 ± 8.73
Child–Pugh classification	
A	155 (36.90%)
B	178 (42.38%)
C	87 (20.71%)
Hepatitis B virus infected	354 (84.3%)
ALT level before LT (U/L)	38 (23.25–68.75)
AST level before LT (U/L)	50 (35–114)
Operation time (min)	460.34 ± 107.69
Intraoperative blood loss (ml)	1,450 (800–2,000)
Intraoperative PRBC transfusions (unit)	4 (2–7)
anhepatic period (min)	57.21 ± 30.30
Peak 7-day postoperative ALT level (U/L)	653 (345.25–1,023.5)
Peak 7-day postoperative AST level (U/L)	435 (232.75–993.25)
EAD	151 (36.0%)
Peak 7-day postoperative Bilirubin level (umol/L)	105.45 (60.235–174.075)
Peak 7-day postoperative Creatinine level (umol/L)	89.0 (72.0–122.75)
Peak 7-day postoperative INR level	1.47 (1.31–1.7)
Biliary complication	34 (8.1%)
Acute rejection	18 (4.3%)
Exceeding Milan criteria^a^	281 (66.9%)
Previous TACE	151 (36.0%)
Previous radiofrequency ablation	98 (23.3%)
Both TACE &radiofrequency ablation	173 (47.3%)
Hepatectomy before LT	60 (14.3%)
AFP (ng/ml)	29.17 (5.26–541.65)
Tumor size (mm)	53.21 ± 40.23
Poor tumor differentiation	120 (28.6%)
Microscopic vascular invasion	110 (26.2%)
Presence of a satellite nodule	259 (61.7%)
HCC recurrence	133 (31.7%)
Recurrence time (m)	16 (9–32)

**Notes.**

a Based on clinical staging.

Abbreviations DCD donation after circulatory death AFP indicates serum *α*-fetoprotein TACE transarterial chemoembolization MELD model for end-stage liver disease EAD early allograft dysfunction LT liver transplant HCC hepatocellular carcinoma PRBC packed red blood cells AST serum aspartate aminotransferase ALT serum alanine aminotransferase INR international normalized ratio

### Effects of CIT on IRI

**Figure 1:**
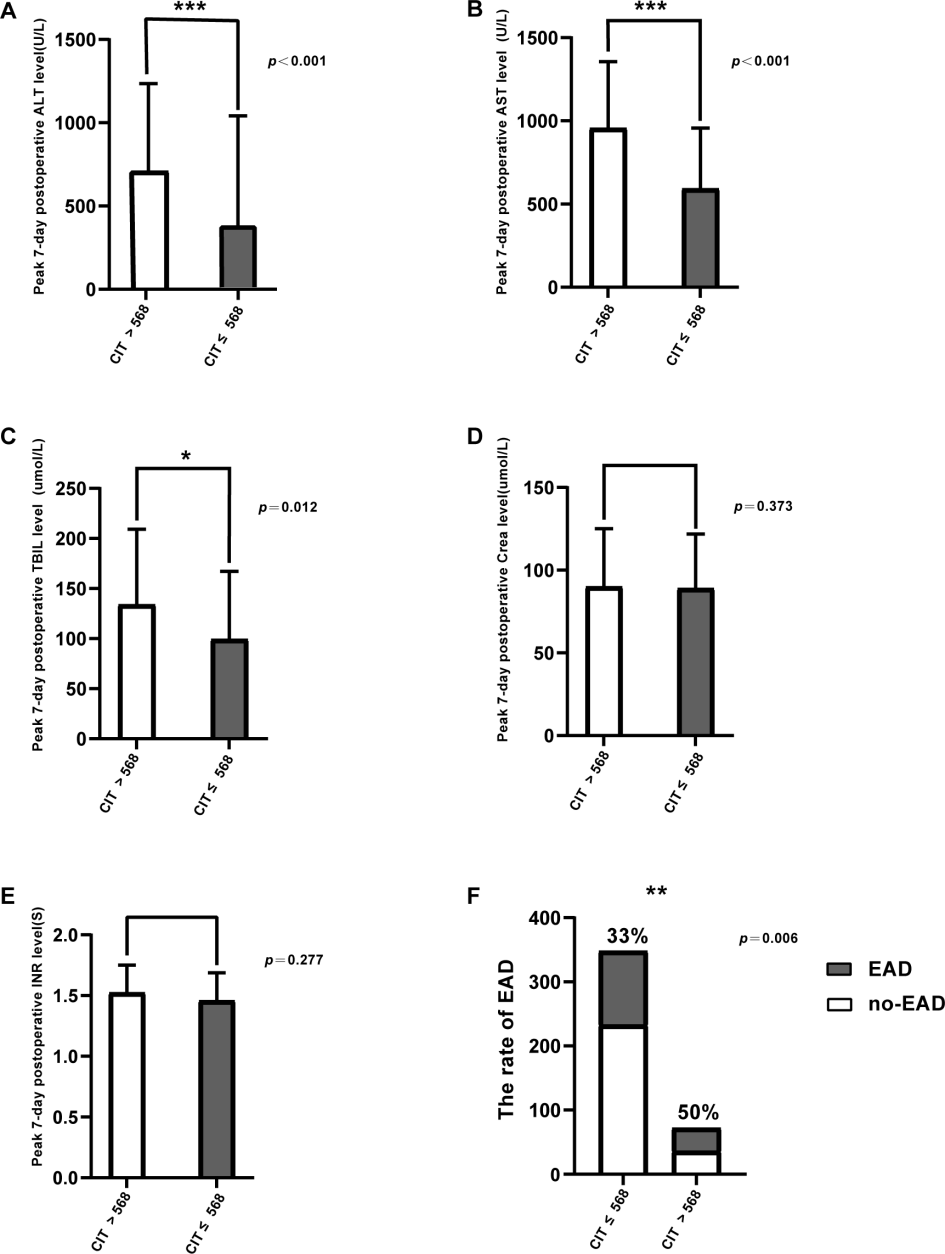
Comparison of posttransplant outcomes by CIT. (A) Peak 7-day postoperative serum alanine aminotransferase (ALT) level (U/L), *p* <0.001. (B) Peak 7-day postoperativeserum aspartate aminotransferase (AST) level (U/L), *p* < 0.001. (C) Peak 7-day postoperative bilirubin level (umol/L), *p* = 0.012. (D) Peak 7-day postoperative creatinine level (umol/L), *p* = 0.373. (E) Peak 7-day postoperative international normalized ratio (INR) level, *p* = 0.277. (F) The incidence of early allograft dysfunction (EAD), *p* = 0.006. * *p* < 0.05, ** *p* < 0.01, *** *p* < 0.001.

We grouped the patients into two: CIT > 568 min and CIT ≤ 568 min according to the optimal cutoff value of the ROC, peak ALT/AST levels, and total bilirubin on POD7. The differences in the rate of EAD was statistically significant (Table 2). Patients with CIT > 568 min showed significantly higher AST, ALT, and TB levels after LT (AST, 712.5 *vs.* 390 U/L, *p* < 0.001; ALT, 957 *vs.* 593 U/L, *p* < 0.001; TB, 134.05 *vs.* 99.5 umol/L, *p* = 0.012; Figs. 1A–1C). However, no statistical differences were found in Crea level and INR level between the two groups (*p* = 0.373, *p* = 0.277, respectively, Figs. 1D, 1E). The EAD incidence in the group with CIT > 568 min was 50%, which was significantly higher than in the group with CIT ≤ 568 min (33.0%) (*p* = 0.006; Fig. 1F). The incidence of biliary complications was 5.6% in the CIT > 568 min group, and 8.6% in CIT ≤ 568 min group. The statistical results also demonstrated that prolonged CIT resulted in more fresh frozen plasma (FFP) infusion during the operation (*p* = 0.02) and longer postoperative respiratory support time (*p* = 0.045).

**Table 2:** Differences in factors represent IRI in clinic between CIT>568 min and CIT ≤568 min.

	CIT > 568(*n* = 72)	CIT ≤568(*n* = 348)	*P*
Peak 7-day postoperative ALT level (U/L)	957 (552.75–1,355.5)	593 (322.5–957.1)	0.000
Peak 7-day postoperative AST level (U/L)	712.5 (381–1,374.25)	390 (219.5–917.5)	0.000
Peak 7-day postoperative TBIL level (umol/L)	134.05 (72.525–209.35)	99.5 (57.375–167.225)	0.012
Peak 7-day postoperative Crea level (umol/L)	90 (74–125)	89 (71–121.75)	0.373
Peak 7-day postoperative INR level (S)	1.525 (1.31–1.75)	1.46 (1.3125–1.6875)	0.277
Biliary complication	4 (5.6%)	30 (8.6%)	0.385
Acute rejection	3 (4.2%)	15 (4.3%)	0.956
EAD	36 (50%)	115 (33.0%)	0.006
Postoperative respiratory support (hour)	20 (12–45)	16 (11–28)	0.045
Infusion of FFP (Unit)	7 (5–12)	6.5 (4–9)	0.02

**Notes.**

Abbreviations AST serum aspartate aminotransferase ALT serum alanine aminotransferase TBIL total bilirubin Crea creatinine INR international normalized ratio EAD early allograft dysfunction FFP fresh frozen plasma

### Risk factors for HCC recurrence

HCC recurrence was observed in 133 patients (31.7%), with the median time to recurrence was 16 months (interquartile range: 9 months-32 months). We compared donor and recipient factors between patients with HCC recurrence (*n* = 133) and those without (*n* = 287). Our findings revealed that in the recurrent group, the proportion of patients with CIT ¿ 568 min significantly increased (26.32% *vs.* 12.89%, *p* = 0.001) (Table 3). Additionally, donors in the recurrence group had significantly higher AST levels before harvest (64 *vs.* 60 U/L, *p* = 0.007), and recipients were younger (50.82 ± 10.93 *vs.* 52.95 ± 9.8 years, *p* = 0.046). There was also a significantly higher rate of male donors in the recurrence group (59.4% *vs.* 48.8%, *p* = 0.043), but a lower rate of diabetes among recipients (7.5% *vs.* 15.3%, *p* = 0.026). Recipients in the recurrence group had a higher MELD score (15.287 ± 9.48 *vs.* 14.233 ± 8.36, *p* = 0.023), and significantly higher TBIL levels before LT (32.3 *vs.* 27.4 umol/L, *p* = 0.002). Furthermore, the recurrence group had a significantly higher percentage of patients exceeding the Milan criteria (79.7% *vs.* 61.0%, *p* = 0.000), larger tumor size (66.96 ± 45.24 *vs.* 46.83 ± 36.02 mm, *p* = 0.000), poorer tumor differentiation (46.6% *vs.* 28.6%, *p* = 0.006), and a higher percentage of microscopic vascular invasion (46.6% *vs.* 16.7%, *p* = 0.000). Lastly, the proportion of recipients with preoperative AFP ≥ 400 ng/ml was significantly higher in the recurrence group (42.9% *vs.* 20.2%, *p* = 0.000) (Table 3).

**Table 3:** Differences in donor factors and recipient factors variables between patients with (*n* = 133) and without (*n* = 287) HCC recurrence.

	HCC recurrence (*n* = 133)	No HCC recurrence (*n* = 287)	*P*
Donor Factors			
Age (years)	37.05 ± 14.25	36.95 ± 14.43	0.946
Body mass index	22.04 ± 2.94	23.37 ± 22.04	0.492
Gender (male)	115 (86.5%)	253 (88.2%)	0.626
Warm ischemia time (minutes) (DCD donor)	5 (3.5–7)	6 (5–10)	0.291
Cold ischemia time >568 (min)	35 (26.32%)	37 (12.89%)	0.001
Sodium level before harvest (mmol/L)	151.24 ± 15.44	154.94 ± 87.03	0.615
ALT level before harvest (U/L)	39 (26–73)	52 (27–89.11)	0.219
AST level before harvest (U/L)	64 (39–130.5)	60 (30–106.99)	0.007
TBIL level before harvest (umol/L)	22.73 ± 17.67	24.07 ± 22.32	0.543
DCD	30 (22.6%)	49 (17.1%)	0.181
Recipient Factors			
Age (years)	50.82 ± 10.93	52.95 ± 9.8	0.046
Body mass index (kg/m^2^)	23.68 ± 3.27	23.07 ± 3.31	0.079
Gender (male)	79 (59.4%)	140 (48.8%)	0.043
Complications			
Hypertension	12 (9.0%)	44 (15.3%)	0.077
Diabetes	10 (7.5%)	44 (15.3%)	0.026
Coronary heart disease	3 (2.3%)	6 (2.1%)	0.913
MELD	15.287 ± 9.48	14.233 ± 8.36	0.023
ALT level before LT (U/L)	39 (26–73)	37 (22–67)	0.544
AST level before LT (U/L)	64 (39–130)	46 (33–98)	0.868
TBIL level before LT (umol/L)	32.3 (16.15–119.6)	27.4 (17.1–50.9)	0.002
Operation time (min)	467.77 ± 114.71	456.89 ± 104.31	0.687
Intraoperative blood loss (ml)	1,500 (800–2,300)	1,200 (800–2,000)	0.503
Intraoperative PRBC transfusions (unit)	4 (2–8)	4 (2–6)	0.391
anhepatic period (min)	60.29 ± 45.5	55.79 ± 19.58	0.157
Infusion of FFP (Unit)	6.5 (4.5–9.3)	6.5 (4–9)	0.399
Peak 7-day postoperative ALT level (U/L)	723 (336.5–1,142)	647 (346–976)	0.304
Peak 7-day postoperative AST level (U/L)	444 (236–947.5)	422 (231–1,002.51)	0.939
Peak 7-day postoperative Tbil level (U/L)	108 (56.05–180.65)	104.3 (62.9–170.5)	0.417
Peak 7-day postoperative Creatinine level (umol/L)	85 (68–113.5)	90 (73–125)	0.117
Peak 7-day postoperative INR level	1.46 (1.30–1.67)	1.48 (1.32–1.71)	0.326
EAD	46 (34.6%)	105 (36.6%)	0.691
Biliary complication	11 (8.3%)	23 (8.0%)	0.928
Acute rejection	6 (4.5%)	12 (4.2%)	0.877
Exceeding Milan criteria	106 (79.7%)	175 (61.0%)	0.000
Previous TACE	48 (36.1%)	103 (35.9%)	0.832
Previous radiofrequency ablation	34 (25.6%)	64 (22.3%)	0.509
Hepatectomy before LT	20 (15.0%)	40 (13.9%)	0.917
AFP (ng/ml) ≥400	57 (42.9%)	58 (20.2%)	0.000
Tumor size (mm)	66.96 ± 45.24	46.83 ± 36.02	0.000
Poor tumor differentiation	56 (42.1%)	82 (28.6%)	0.006
Microscopic vascular invasion	62 (46.6%)	48 (16.7%)	0.000

**Notes.**

Abbreviations DCD donation after circulatory death AFP indicates serum *α*-fetoprotein TACE transarterial chemoembolization MELD model for end-stage liver disease EAD early allograft dysfunction LT liver transplant HCC hepatocellular carcinoma AST serum aspartate aminotransferase ALT serum alanine aminotransferase TBIL total bilirubin PRBC packed red blood cells INR international normalized ratio FFP fresh frozen plasma

Next,the factors that differed between the two groups were analyzed in a multivariate analysis by using logistic regression. Cold ischemia time > 568 min (OR, 2.406; 95% CI [1.371–4.220]; *p* = 0.002), Exceeding Milan criteria (OR, 2.135; 95% CI [1.269–3.590]; *p* = 0.004), AFP (ng/ml) ≥400 (OR, 2.195; 95% CI [1.360–3.543]; *p* = 0.001), poor tumor differentiation (OR, 1.612; 95% CI [1.009–2.577]; *p* = 0.046) were independent predictors of recurrence (Table 4).

**Table 4:** Multivariate analysis for risk factors of HCC recurrence.

	OR (95% CI)	*P*value^*^
Donor characteristics		
Cold ischemia time >568	2.406 (1.371–4.220)	0.002
AST level before harvest (U/L)	1.000 (0.999–1.001)	0.654
Recipient characteristics		
Age (years)	1.018 (0.996–1.041)	0.116
Gender (male)	1.300 (0.668–2.529)	0.440
Diabetes	0.706 (0.338–1.473)	0.353
MELD	0.994 (0.969–1.019)	0.615
TBIL level before LT (umol/L)	1.000 (0.997–1.002)	0.792
Exceeding Milan criteria	2.135 (1.269–3.590)	0.004
AFP (ng/ml) ≥400	2.195 (1.360–3.543)	0.001
Tumor numbers >3	NA※	NA※
Tumor size (mm)>50	NA※	NA※
Poor tumor differentiation	1.612 (1.009–2.577)	0.046
Microscopic vascular invasion	NA※	NA※

**Notes.**

* Logistic regression model analysis.

NA※, Tumor size, number of tumor, and MVI were not included regression model, as they are included in Milan criteria.

Abbreviations MELD model for end-stage liver disease HCC hepatocellular carcinoma AST serum aspartate aminotransferase TBIL total bilirubin

### CIT > 568 min is an independent risk factor for DFS but not for OS

**Figure 2:**
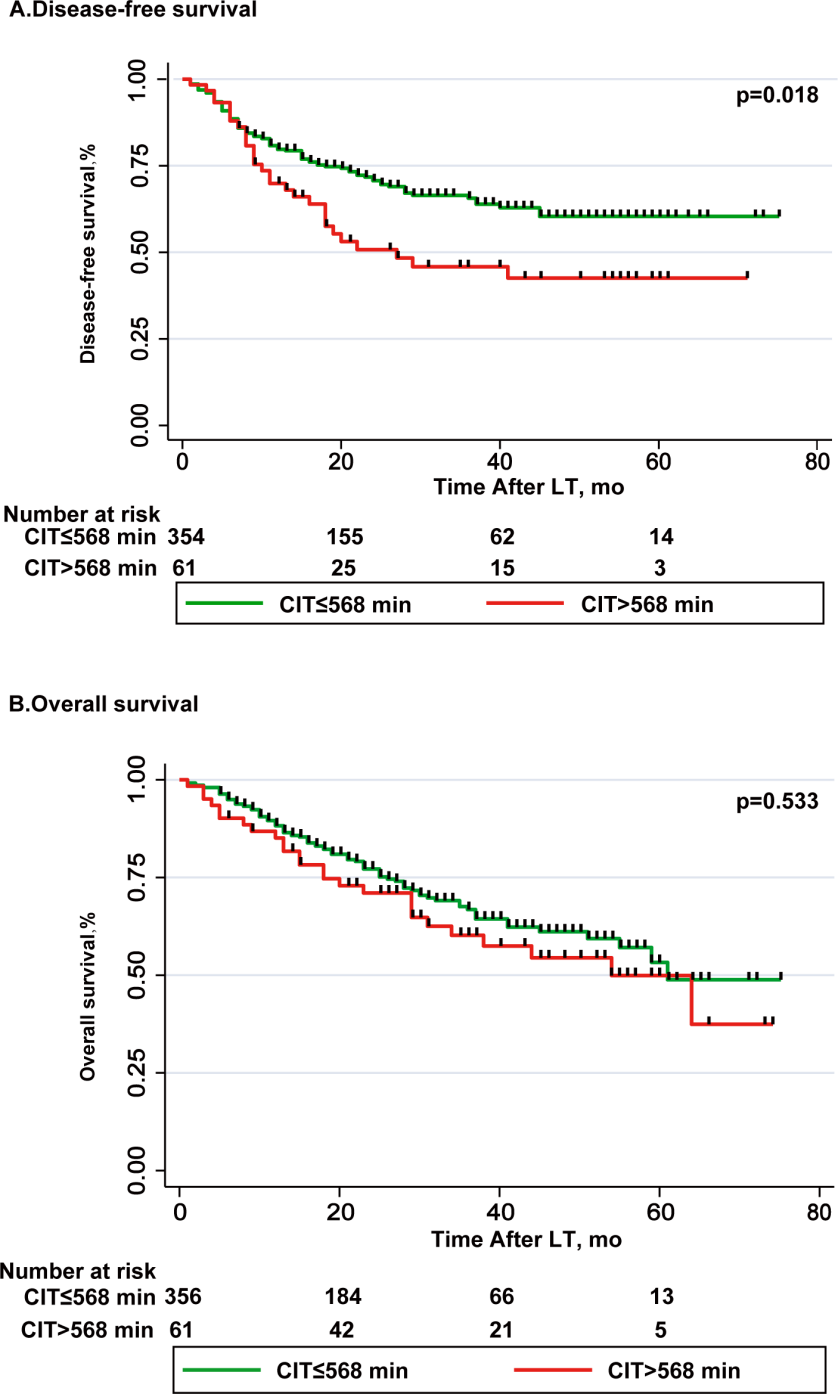
Comparison of disease-free survival and overall survival in CIT ≤568 min group and CIT >568 min group. (A) Disease-free survival (DSF) rate was significantly higher after LT with CIT ≤ 568 min as compared to LT with CIT >568 min, *p* = 0.018. (B) Overall survival (OS) rate was lower after LT with CIT >568 min as compared to LT with CIT ≤ 568 min, difference was not statistically significant, *p* = 0.533.

Table 5 lists the parameters related to risk of disease-free survival (DSF). Multivariate Cox regression analysis showed that the recipient gender (male) (OR, 1.774; 95% CI [1.241–2.536]; *p* = 0.002), AFP ≥ 400 (ng/ml) (OR, 2.032; 95% CI [1.415–2.920]; *p* = 0.009), exceeding Milan criteria (OR, 1.782; 95% CI [1.156–2.747]; *p* = 0.009) and poor tumor differentiation (OR, 1.48; 95% CI [1.041–2.116]; *p* = 0.029) were independent risk factors for DSF (Table 6). DFS rates at 1-and 3-year in CIT > 568 min group were 71.9% and 45.7%, respectively; 79.2% and 66.3%, respectively in CIT ≤568 min group (*p* = 0.018, Fig. 2A). Table 6 lists the parameters related to risk of overall survival (OS), and this is significant in univariate analysis. In multivariate Cox regression analysis, intraoperative PRBC transfusions (unit) (OR, 1.046; 95% CI [1.018–1.076]; *p* = 0.001), AFP ≥ 400 (ng/ml) (OR, 2.42; 95% CI [1.648–3.554]; *p* < 0.001) and poor tumor differentiation (OR, 1.48; 95% CI [1.041–2.116]; *p* = 0.017) were independent risk factors for overall survival (Table 5). OS rates at 1-and 5-year in CIT > 568 min group were 86% and 53%, respectively; 87.4% and 54.1%, respectively in CIT ≤568 min group (*p* = 0.533, Fig. 2B).

**Table 5:** Prognostic factors for disease-free survival on univariate and multivariate analysis.

	Univariate		Multivariate	
Variables	OR (95% CI)	*P* value^*^	OR (95% CI)	*P* value^*^
Donor characteristics				
Age (years)	1.004 (0.992–1.017)	0.485		
Body mass index (kg/m^2^)	0.996 (0.974–1.018)	0.714		
Gender (male)	1.247 (0.821–1.893)	0.3		
Warm ischemia time (minutes)(80)	0.944 (0.844–1.057)	0.318		
Cold ischemia time >568	1.688 (1.146–2.484)	0.008	1.652 (1.111–2.458)	0.013
Sodium level before harvest (mmol/L)	0.999 (0.994–1.004)	0.566		
ALT level before harvest (U/L)	1.001 (1.000–1.002)	0.125		
AST level before harvest (U/L)	1.0 (0.999–1.001)	0.807		
TBIL level before harvest (umol/L)	0.998 (0.989–1.007)	0.651		
DCD	0.866 (0.574–1.309)	0.495		
Recipient characteristics				
Age (years)	0.986 (0.970–1.003)	0.098		
Body mass index (kg/m^2^)	1.046 (0.995–1.101)	0.080		
Gender (male)	0.982 (0.588–1.640)	0.982		
Complications				
Hypertension	1.627 (0.899–2.945)	0.108		
Diabetes	1.951 (0.991–3.839)	0.053		
Coronary heart disease	1.028 (0.327–3.232)	0.963		
MELD	1.022 (1.003–1.041)	0.022	1.013 (0.995–1.032)	0.155
ALT level before LT (U/L)	1.0 (0.999–1.000)	0.544		
AST level before LT (U/L)	1 (1–1)	0.868		
TBIL level before LT (umol/L)	1.002 (1.001–1.003)	0.052		
Operation time (min)	1.000 (0.999–1.002)	0.885		
Intraoperative blood loss (ml)	1 (1–1)	0.687		
Intraoperative PRBC transfusions (unit)	1.013 (0.987–1.042)	0.391		
anhepatic period (min)	1.001 (0.996–1.005)	0.817		
Peak 7-day postoperative ALT level (U/L)	1 (1–1)	0.256		
Peak 7-day postoperative AST level (U/L)	1 (1–1)	0.205		
EAD	1.219 (0.85–1.749)	0.282		
Peak 7-day postoperative Creatinine level (umol/L)	1.0 (0.998–1.002)	0.923		
Peak 7-day postoperative INR level	0.999 (0.997–1.001)	0.307		
	0.872 (0.602–1.259)	0.872		
Biliary complication	1.065 (0.574–1.974)	0.842		
Acute rejection	1.298 (0.572–2.950)	0.533		
Infusion of FFP (Unit)	0.999 (0.993–1.004)	0.611		
Exceeding Milan criteria^a^	2.085 (1.366–3.184)	0.001	1.742 (1.129–2.687)	0.012
Previous TACE	1.039 (0.729–1.481)	0.832		
Previous radiofrequency ablation	0.877 (0.593–1.296)	0.509		
Hepatectomy before LT	1.026 (0.637–1.651)	0.917		
AFP (ng/ml) ≥400	2.953 (2.093–4.166)	0.000	2.410 (1.679–3.461)	0.000
Tumor numbers >3	1.668 (1.185–2.347)	0.003	NA※	NA※
Tumor size (mm)>50	1.518 (1.078–2.136)	0.017	NA※	NA※
Poor tumor differentiation	1.749 (1.236–2.475)	0.002	1.547 (1.086–2.203)	0.016
Microscopic vascular invasion	3.564 (2.524–5.033)	0.000	NA※	NA※

**Notes.**

* Cox’s proportional regression analysis.

NA※, Tumor size, number of tumor, and MVI were not included to avoid colinearity, as they are included in Milan criteria.

Abbreviations DCD donation after circulatory death AFP indicates serum *α*-fetoprotein TACE transarterial chemoembolization MELD model for end-stage liver disease EAD early allograft dysfunction LT liver transplant HCC hepatocellular carcinoma PRBC packed red blood cells AST serum aspartate aminotransferase ALT serum alanine aminotransferase TBIL total bilirubin INR international normalized ratio FFP fresh frozen plasma

**Table 6:** Prognostic factors for overall survival on univariate and multivariate analysis.

Variables	Univariate		Multivariate	
	OR (95% CI)	*P*value^*^	OR (95% CI)	*P*value^*^
Donor characteristics				
Age (years)	1.01(0.997–1.023)	0.137		
Body mass index (kg/m^2^)	1 (0.985–1.015)	0.986		
Gender (male)	1.069 (0.701–1.629)	0.757		
Warm ischemia time (minutes)(80)	0.913 (0.823–1.012)	0.084		
Cold ischemia time >568	1.143 (0.751–1.738)	0.533		
Sodium level before harvest (mmol/L)	0.997 (0.989–1.006)	0.559		
ALT level before harvest (U/L)	1.001 (1.000–1.002)	0.046	NA#	NA#
AST level before harvest (U/L)	1.0 (1.0–1.001)	0.191		
TBIL level before harvest (umol/L)	1.005 (0.998–1.013)	0.143		
DCD	0.866 (0.574–1.309)	0.495		
Recipient characteristics				
Age (years)	1.002 (0.985–1.020)	0.789		
Body mass index (kg/m^2^)	1.021 (0.966–1.078)	0.467		
Gender (male)	0.962 (0.585–1.581)	0.877		
Complications				
Hypertension	1.315 (0.805–2.148)	0.273		
Diabetes	1.187 (0.654–2.155)	0.572		
Coronary heart disease	1.316 (0.325–5.336)	0.7		
MELD	1.028 (1.009–1.047)	0.004	1.016 (0.996–1.036)	0.114
ALT level before LT (U/L)	1.0	0.510		
AST level before LT (U/L)	1	0.858		
TBIL level before LT (umol/L)	1.002 (1.001–1.003)	0.000		
Operation time (min)	1.001 (0.999–1.002)	0.45		
Intraoperative blood loss (ml)	1 (1–1)	0.084		
Intraoperative PRBC transfusions (unit)	1.036 (1.010–1.063)	0.007	1.048 (1.019–1.078)	0.001
anhepatic period (min)	1.00 (0.996–1.005)	0.863		
Peak 7-day postoperative ALT level (U/L)	1 (1–1)	0.000	NA#	NA#
Peak 7-day postoperative AST level (U/L)	1 (1–1)	0.034	NA#	NA#
EAD	1.172 (0.819–1.677)	0.385		
Peak 7-day postoperative Bilirubin level (umol/L)	1.001 (1.000–1.003)	0.104		
Peak 7-day postoperative Creatinine level (umol/L)	0.999 (0.997–1.002)	0.610		
Peak 7-day postoperative INR level	0.975 (0.857–1.110)	0.706		
Biliary complication	1.045 (0.547–1.996)	0.895		
Acute rejection	1.230 (0.501–3.018)	0.651		
Infusion of FFP (Unit)	1 (0.999–1.001)	0.938		
Exceeding Milan criteria^a^	1.8 (1.174–2.759)	0.007	1.388(0.895–2.154)	0.143
Previous TACE	1.419 (0.962–2.092)	0.078		
Previous radiofrequency ablation	1.241 (0.794–1.940)	0.344		
Hepatectomy before LT	1.25 (0.727–2.147)	0.42		
AFP (ng/ml) ≥400	3.195 (2.239–4.558)	0.000	2.915 (1.982–4.287)	0.000
Tumor numbers >3	1.819 (1.276–2.594)	0.001	NA※	NA※
Tumor size (mm)>50	1.557 (1.093–2.218)	0.014	NA※	NA※
Poor tumor differentiation	1.921 (1.343–2.746)	0.000	1.577 (1.083–2.298)	0.018
Microscopic vascular invasion	3.038 (2.115–4.362)	0.000	NA※	NA※

**Notes.**

* Cox’s proportional regression analysis.

NA# not applicable.

NA※, Tumor size, number of tumor, and MVI were not included to avoid colinearity, as they are included in Milan criteria.

Abbreviations DCD donation after circulatory death AFP indicates serum *α*-fetoprotein TACE transarterial chemoembolization MELD model for end-stage liver disease EAD early allograft dysfunction LT liver transplant HCC hepatocellular carcinoma PRBC packed red blood cells AST serum aspartate aminotransferase ALT serum alanine aminotransferase TBIL total bilirubin INR international normalized ratio FFP fresh frozen plasma

## Discussion

The quality of donor grafts is one of the most important factors affecting ischemia-reperfusion injury of LT. Moreover, ischemic time, cold ischemic time, and warm ischemic time are vital indices for evaluating the quality of the donor’s liver. This study showed that a prolongation of CIT elevated the AST, ALT, and bilirubin levels to 7 days after LT. Unsurprisingly, the EAD incidence was significantly higher in the CIT > 568 min group. This result agrees with the findings of a previous study regarding ischemia-reperfusion and EAD (Ito et al., 2021). In addition, more intraoperative infusion of FFP and longer respiratory support time suggested a worse recovery in CIT > 568 min group. Currently, LT is an optional treatment for HCC based on the tumor characteristics. However, research on donor-related factors and their impact on tumor recurrence is still insufficient. Numerous studies have argued that liver ischemia is a risk factor for liver cancer recurrence (Yoshimoto et al., 2012; Yang et al., 2019; Kornberg et al., 2015b).

This study demonstrated that the CIT of liver grafts is an independent risk factor for the tumor recurrence after LT in patients with HCC. Another study of 103 LT patients reported that prolonged mean CIT and WIT promoted the risk of HCC recurrence (Fisher et al., 2007). The survival rate of patients and grafts was lower in HCC recipients who received DCD allografts which suggested that IRI injury had a greater impact on HCC recipients, despite the lack of HCC recurrence data (Kornberg et al., 2015a). A large retrospective studyused the data of 9,724 patients with HCC who received an LT to conclude that prolonged donor warm ischemia time was a risk factor for HCC recurrence (Croome et al., 2013). A single-center study elaborated that AST ≥ 1,896 U/L was a risk factor for HCC recurrence after LT with DBD grafts (Duvoux et al., 2012). Moreover, donor age > 60 years and donor WIT were the risk factors for increased HCC recurrence (Orci et al., 2018). Thus, these organs are susceptible to intensified IRI, which may lead to higher rates of HCC recurrence after LT.

In our study, 30 of 80 patients, who received a liver graft from DCD donors, experienced HCC recurrence. Compared with the DBD group, the HCC recurrence rate was not statistically significant (*p* = 0.642; univariate Cox’s regression analysis, the reference to the data file as a Supplementary File), which might be due to the small sample size.

Although numerous studies have reported that IRI could elevate the risk of HCC recurrence (Grat et al., 2018; Orci et al., 2018; Croome et al., 2015), the mechanisms are not yet fully understood owing to the complex network of cellular and molecular interactions. Moreover, the mechanism of injury caused by different forms of IRI varies, and the alteration of the hepatic microenvironment could create a “fertile soil” for tumor cells. For instance, sinusoidal endothelial cells (SECs) are vulnerable to the IRI, particularly during cold ischemia, which may be the risk factor for endothelial cell swelling, unbalanced vasoconstriction, and neutrophil plugging (Tejima et al., 2004). In addition, IRI can break the barrier between hepatocytes and the blood, making molecules and cells spread easily. Microcirculatory disturbances lead to tissue ischemia and hypoxia. It also activates the HIF-1 *α* pathway, which has been recognized as a vascular endothelial growth factor associated with metastatic disease (Semenza, 2012). IRI-induced inflammatory cytokines, tumor necrosis factor- *α*, or interleukin-1 may promote the metastasis of different cancer cells through the expression of adhesion molecules (such as E-selectin, ICAM-1, and VCM-1), acting as mediators of tumor growth (Yoshimoto et al., 2012; Reina & Espel, 2017; Kong et al., 2018).

Damage-associated molecular patterns (DAMPs) are other important mechanisms of IRI, promoting tumor progression, which has been released by injured hepatic cells and may perpetuate the non-infectious sterile inflammatory response. The DAMPs released into the blood can cause innate immune activation and robust hepatocellular injury. For instant, high-mobility group protein B1 (HMGB1), which is the most frequently encountered DAMP in cells undergoing stress, could translocate from the nucleus to the cytosol, bind to mitochondrial DNA (mtDNA), and be released by damaged mitochondria during hypoxia (Srikrishna & Freeze, 2009). The complex of HMGB1 and mtDNA subsequently activates the TLR9 signaling pathways to promote tumor cell proliferation (Liu et al., 2015). Tohme et al. (2017) found that upregulated HMGB1 could enhance mitochondrial biogenesis in HCC cancer cells and promote tumor survival and proliferation.

Many marginal donor livers are used clinically owing to the lack of a donation pool. This issue can lead to more serious IRI damage, subsequent complications, and HCC recurrence after transplantation. Thus, evaluating liver function and repairing the liver *in vitro* is vital to reducing complications after transplantation. Traditional static cold storage (SCS) may be impossible. With the progress of perfusion technology and the upgrading of perfusion machines, mechanical perfusion has been applied significantly for marginal liver donation. The process is effective in refurnishing energy stores during preservation to resist impending ischemia-reperfusion injury after organ implant. In addition, *ex situ* organ perfusion simulates physiological blood reflow, reflects the quality of organs, and provides an objective functional evaluation for transplant surgeons. Currently, hypothermic (hypothermic oxygenated perfusion, HOPE) and normothermic (normothermic machine perfusion, NMP) and their combination are widely used machine perfusion approaches (Karangwa et al., 2016). Several clinical trials have confirmed that the HOPE technique offers promising advantages compared with SCS in patients whose organs are transplanted from DCD donors (Karangwa et al., 2016; Schlegel et al., 2019). Another group reported an encouraging result, revealing the incidence of symptomatic non-anastomotic biliary strictures in dual hypothermic oxygenated machine perfusion (D-HOPE), which was perfused through the hepatic artery and portal vein, showing that liver graft volumes in 78 patients were significantly lower after LT than in the SCS group (78 patients) after LT from DCD donors (Schlegel et al., 2013). Ischemia-free organ transplantation (IFOT) eliminates the ischemia and hypoxia of the organ by uninterrupted NMP during the procurement and implant periods. A retrospective study also demonstrated that patients who underwent the IFOT had significant higher DFS rates at 1 and 3 years than the SCS group after LT in recipients with HCC (Tang et al., 2021).

The disadvantages of ischemia-reperfusion in treating hepatocellular carcinoma have been documented. Compared with hepatectomy, LT is more vulnerable to IRI because of the sophisticated segments and procedures. However, the assessment of the patients’ oncological risk profile following LT is limited to the clinical characteristics and biological features of carcinoma. CIT is the easiest to evaluate compared with complicated biological and histological tests; although CIT is not the most direct evidence of IRI. Our results show that prolonged CIT can significantly increase the peak level of ALT, AST, TBIL, and the incidence of EAD in patients after LT.

Although several studies had found that prolonged cold ischemia time promotes recurrence after LT for HCC, their research did not cover East Asian people. Our study retrospectively analyzed 420 Chinese HCC patients with LT, demonstrated that CIT > 568 min was an independent risk factor for recurrence after LT in patients with HCC, which move forward the CIT window that warns of HCC recurrence. Therefore, the significance of CIT in patients undergoing LT for HCC should be re-evaluated.

Our study is a single-center retrospective study, which is its limitation. Future studies must conduct multicenter, prospective cohort studies and explore the biological mechanism behind IRI and tumor recurrence, which may guide the selection of grafts for HCC patients undergoing LT, and provide therapeutic targets for reducing ischemia-reperfusion and recurrence after LT.

## Conclusion

In conclusion, our study confirms the harmful effect of cold ischemia time on the prognosis of liver transplantation patients with HCC. Cold ischemia time should be reconsidered when evaluating patients with HCC on the waiting list. Shortening the cold ischemia time should be one of the effective strategies to improve the prognosis, especially for those with relatively low tumor load and long expected survival.

## Supplemental Information

10.7717/peerj.18126/supp-1 Supplemental Information 1 Raw data

## Additional Information and Declarations

### Data Availability

The following information was supplied regarding data availability: The raw data is available in the Supplementary File.

## References

[ref-1] Adam R, Karam V, Cailliez V, O’Grady JG, Mirza D, Cherqui D, Klempnauer J, Salizzoni M, Pratschke J, Jamieson N, Hidalgo E, Paul A, Andujar RL, Lerut J, Fisher L, Boudjema K, Fondevila C, Soubrane O, Bachellier P, Pinna AD, Berlakovich G, Bennet W, Pinzani M, Schemmer P, Zieniewicz K, Romero CJ, De Simone P, Ericzon BG, Schneeberger S, Wigmore SJ, Prous JF, Colledan M, Porte RJ, Yilmaz S, Azoulay D, Pirenne J, Line PD, Trunecka P, Navarro F, Lopez AV, De Carlis L, Pena SR, Kochs E, Duvoux C, all the other 126 contributing centers (www.eltr.org), The European Liver and Intestine Association (ELITA) (2018). 2018 Annual report of the European Liver Transplant Registry (ELTR) - 50-year evolution of liver transplantation. Transplant International.

[ref-2] Busuttil RW, Farmer DG, Yersiz H, Hiatt JR, McDiarmid SV, Goldstein LI, Saab S, Han S, Durazo F, Weaver M, Cao C, Chen T, Lipshutz GS, Holt C, Gordon S, Gornbein J, Amersi F, Ghobrial RM (2005). Analysis of long-term outcomes of 3200 liver transplantations over two decades: a single-center experience. Annals of Surgery.

[ref-3] Cassuto JR, Patel SA, Tsoulfas G, Orloff MS, Abt PL (2008). The cumulative effects of cold ischemic time and older donor age on liver graft survival. Journal of Surgical Research.

[ref-4] Chini CCS, Zeidler JD, Kashyap S, Warner G, Chini EN (2021). Evolving concepts in NAD(+) metabolism. Cell Metabolism.

[ref-5] Choi GH, Kim GI, Yoo JE, Na DC, Han DH, Roh YH, Park YN, Choi JS (2015). Increased expression of circulating cancer stem cell markers during the perioperative period predicts early recurrence after curative resection of hepatocellular carcinoma. Annals of Surgical Oncology.

[ref-6] Croome KP, Lee DD, Burns JM, Musto K, Paz D, Nguyen JH, Perry DK, Harnois DM, Taner CB (2015). The use of donation after cardiac death allografts does not increase recurrence of hepatocellular carcinoma. American Journal of Transplantation.

[ref-7] Croome KP, Wall W, Chandok N, Beck G, Marotta P, Hernandez-Alejandro R (2013). Inferior survival in liver transplant recipients with hepatocellular carcinoma receiving donation after cardiac death liver allografts. Liver Transplantation.

[ref-8] Duvoux C, Roudot-Thoraval F, Decaens T, Pessione F, Badran H, Piardi T, Francoz C, Compagnon P, Vanlemmens C, Dumortier J, Dharancy S, Gugenheim J, Bernard PH, Adam R, Radenne S, Muscari F, Conti F, Hardwigsen J, Pageaux GP, Chazouilleres O, Salame E, Hilleret MN, Lebray P, Abergel A, Debette-Gratien M, Kluger MD, Mallat A, Azoulay D, Cherqui D, Liver Transplantation French Study G (2012). Liver transplantation for hepatocellular carcinoma: a model including alpha-fetoprotein improves the performance of Milan criteria. Gastroenterology.

[ref-9] Fisher RA, Kulik LM, Freise CE, Lok AS, Shearon TH, Brown Jr RS, Ghobrial RM, Fair JH, Olthoff KM, Kam I, Berg CL, AAS Group (2007). Hepatocellular carcinoma recurrence and death following living and deceased donor liver transplantation. American Journal of Transplantation.

[ref-10] Grat M, Krawczyk M, Wronka KM, Stypulkowski J, Lewandowski Z, Wasilewicz M, Krawczyk P, Grat K, Patkowski W, Zieniewicz K (2018). Ischemia-reperfusion injury and the risk of hepatocellular carcinoma recurrence after deceased donor liver transplantation. Scientific Reports.

[ref-11] Ito T, Naini BV, Markovic D, Aziz A, Younan S, Lu M, Hirao H, Kadono K, Kojima H, Di Norcia 3rd J, Agopian VG, Yersiz H, Farmer DG, Busuttil RW, Kupiec-Weglinski JW, Kaldas FM (2021). Ischemia-reperfusion injury and its relationship with early allograft dysfunction in liver transplant patients. American Journal of Transplantation.

[ref-12] Kalisvaart M, Croome KP, Hernandez-Alejandro R, Pirenne J, Cortes-Cerisuelo M, Minambres E, Abt PL (2021). Donor warm ischemia time in DCD liver transplantation-working group report from the ILTS DCD, liver preservation, and machine perfusion consensus conference. Transplantation.

[ref-13] Karangwa SA, Dutkowski P, Fontes P, Friend PJ, Guarrera JV, Markmann JF, Mergental H, Minor T, Quintini C, Selzner M, Uygun K, Watson CJ, Porte RJ (2016). Machine perfusion of donor livers for transplantation: a proposal for standardized nomenclature and reporting guidelines. American Journal of Transplantation.

[ref-14] Kong DH, Kim YK, Kim MR, Jang JH, Lee S (2018). Emerging roles of vascular cell adhesion molecule-1 (VCAM-1) in immunological disorders and cancer. International Journal of Molecular Sciences.

[ref-15] Kornberg A, Witt U, Kornberg J, Friess H, Thrum K (2015a). Extended ischemia times promote risk of HCC recurrence in liver transplant patients. Digestive Diseases and Sciences.

[ref-16] Kornberg A, Witt U, Kornberg J, Friess H, Thrum K (2015b). Treating ischaemia-reperfusion injury with prostaglandin E1 reduces the risk of early hepatocellular carcinoma recurrence following liver transplantation. Alimentary Pharmacology & Therapeutics.

[ref-17] Liu Y, Yan W, Tohme S, Chen M, Fu Y, Tian D, Lotze M, Tang D, Tsung A (2015). Hypoxia induced HMGB1 and mitochondrial DNA interactions mediate tumor growth in hepatocellular carcinoma through Toll-like receptor 9. Journal of Hepatology.

[ref-18] Mehta N, Heimbach J, Lee D, Dodge JL, Harnois D, Burns J, Sanchez W, Roberts JP, Yao FY (2017). Wait time of less than 6 and greater than 18 months predicts hepatocellular carcinoma recurrence after liver transplantation: proposing a wait time sweet spot. Transplantation.

[ref-19] Nagai S, Yoshida A, Facciuto M, Moonka D, Abouljoud MS, Schwartz ME, Florman SS (2015). Ischemia time impacts recurrence of hepatocellular carcinoma after liver transplantation. Hepatology.

[ref-20] Olthoff KM, Kulik L, Samstein B, Kaminski M, Abecassis M, Emond J, Shaked A, Christie JD (2010). Validation of a current definition of early allograft dysfunction in liver transplant recipients and analysis of risk factors. Liver Transplantation.

[ref-21] Orci LA, Lacotte S, Delaune V, Slits F, Oldani G, Lazarevic V, Rossetti C, Rubbia-Brandt L, Morel P, Toso C (2018). Effects of the gut-liver axis on ischaemia-mediated hepatocellular carcinoma recurrence in the mouse liver. Journal of Hepatology.

[ref-22] Pinna AD, Yang T, Mazzaferro V, De Carlis L, Zhou J, Roayaie S, Shen F, Sposito C, Cescon M, Di Sandro S, Yi-Feng H, Johnson P, Cucchetti A (2018). Liver transplantation and hepatic resection can achieve cure for hepatocellular carcinoma. Annals of Surgery.

[ref-23] Reina M, Espel E (2017). Role of LFA-1 and ICAM-1 in cancer. Cancers.

[ref-24] Schlegel A, Graf R, Clavien PA, Dutkowski P (2013). Hypothermic oxygenated perfusion (HOPE) protects from biliary injury in a rodent model of DCD liver transplantation. Journal of Hepatology.

[ref-25] Schlegel A, Muller X, Kalisvaart M, Muellhaupt B, Perera M, Isaac JR, Clavien PA, Muiesan P, Dutkowski P (2019). Outcomes of DCD liver transplantation using organs treated by hypothermic oxygenated perfusion before implantation. Journal of Hepatology.

[ref-26] Semenza GL (2012). Hypoxia-inducible factors in physiology and medicine. Cell.

[ref-27] Srikrishna G, Freeze HH (2009). Endogenous damage-associated molecular pattern molecules at the crossroads of inflammation and cancer. Neoplasia.

[ref-28] Tang Y, Wang T, Ju W, Li F, Zhang Q, Chen Z, Gong J, Zhao Q, Wang D, Chen M, Guo Z, He X (2021). Ischemic-free liver transplantation reduces the recurrence of hepatocellular carcinoma after liver transplantation. Frontiers in Oncology.

[ref-29] Tejima K, Arai M, Ikeda H, Tomiya T, Yanase M, Inoue Y, Nagashima K, Nishikawa T, Watanabe N, Omata M, Fujiwara K (2004). Ischemic preconditioning protects hepatocytes via reactive oxygen species derived from Kupffer cells in rats. Gastroenterology.

[ref-30] Tohme S, Yazdani HO, Liu Y, Loughran P, Van der Windt DJ, Huang H, Simmons RL, Shiva S, Tai S, Tsung A (2017). Hypoxia mediates mitochondrial biogenesis in hepatocellular carcinoma to promote tumor growth through HMGB1 and TLR9 interaction. Hepatology.

[ref-31] Villanueva A (2019). Hepatocellular carcinoma. New England Journal of Medicine.

[ref-32] Yang F, Zhang Y, Ren H, Wang J, Shang L, Liu Y, Zhu W, Shi X (2019). Ischemia reperfusion injury promotes recurrence of hepatocellular carcinoma in fatty liver via ALOX12-12HETE-GPR31 signaling axis. Journal of Experimental & Clinical Cancer Research.

[ref-33] Yoshimoto K, Tajima H, Ohta T, Okamoto K, Sakai S, Kinoshita J, Furukawa H, Makino I, Hayashi H, Nakamura K, Oyama K, Inokuchi M, Nakagawara H, Itoh H, Fujita H, Takamura H, Ninomiya I, Kitagawa H, Fushida S, Fujimura T, Wakayama T, Iseki S, Shimizu K (2012). Increased E-selectin in hepatic ischemia-reperfusion injury mediates liver metastasis of pancreatic cancer. Oncology Reports.

